# The Telomerase Complex Directly Controls Hematopoietic Stem Cell Differentiation and Senescence in an Induced Pluripotent Stem Cell Model of Telomeropathy

**DOI:** 10.3389/fgene.2018.00345

**Published:** 2018-08-29

**Authors:** Shyam Sushama Jose, Federico Tidu, Petra Burilova, Tomas Kepak, Kamila Bendickova, Jan Fric

**Affiliations:** ^1^Cellular and Molecular Immunoregulation Group, Center for Translational Medicine, International Clinical Research Center, St. Anne’s University Hospital Brno, Brno, Czechia; ^2^Department of Biology, Faculty of Medicine, Masaryk University, Brno, Czechia; ^3^Pediatric Oncology Translational Research, International Clinical Research Center, St. Anne’s University Hospital Brno, Brno, Czechia; ^4^Pediatric Hematology and Oncology, The University Hospital Brno, Brno, Czechia

**Keywords:** telomerase imbalance, hematopoiesis, immune function, immunosenescence, iPSC, dyskeratosis congenita, myelopoiesis

## Abstract

Telomeropathies are rare disorders associated with impaired telomere length control mechanisms that frequently result from genetic mutations in the telomerase complex. Dyskeratosis congenita is a congenital progressive telomeropathy in which mutation in the telomerase RNA component (*TERC*) impairs telomere maintenance leading to accelerated cellular senescence and clinical outcomes resembling premature aging. The most severe clinical feature is perturbed hematopoiesis and bone-marrow failure, but the underlying mechanisms are not fully understood. Here, we developed a model of telomerase function imbalance using shRNA to knockdown *TERC* expression in human induced pluripotent stem cells (iPSCs). We then promoted *in vitro* hematopoiesis in these cells to analyze the effects of *TERC* impairment. Reduced *TERC* expression impaired hematopoietic stem-cell (HSC) differentiation and increased the expression of cellular senescence markers and production of reactive oxygen species. Interestingly, telomere length was unaffected in shTERC knockdown iPSCs, leading to conclusion that the phenotype is controlled by non-telomeric functions of telomerase. We then assessed the effects of *TERC*-depletion in THP-1 myeloid cells and again observed reduced hematopoietic and myelopoietic differentiative potential. However, these cells exhibited impaired telomerase activity as verified by accelerated telomere shortening. shTERC-depleted iPSC-derived and THP-1-derived myeloid precursors had lower phagocytic capacity and increased ROS production, indicative of senescence. These findings were confirmed using a BIBR1532 TERT inhibitor, suggesting that these phenotypes are dependent on telomerase function but not directly linked to telomere length. These data provide a better understanding of the molecular processes driving the clinical signs of telomeropathies and identify novel roles of the telomerase complex other than regulating telomere length.

## Introduction

Telomeres are repetitive nucleotide sequences found on the ends of chromosomes that ensure chromosome integrity by protecting chromosome ends from degradation or fusion. The telomere complex machinery is highly active in proliferating and differentiating stem cells (Hiyama and Hiyama, 2007). Telomeres progressively shorten with each cell division as a consequence of the “end replication problem” that telomeres themselves evolved to prevent occurring on the chromosome (Blackburn, 1991; Cawthon et al., 2003). This shortening impairs cellular proliferation and overall cellular regenerative capacity (Flores et al., 2005; Sharpless and DePinho, 2007), and is thus a useful indicator of cellular senescence. As such, links have been made between telomere length, aging, longevity, and aging-associated disorders (von Zglinicki and Martin-Ruiz, 2005; Lehmann et al., 2013; Tian et al., 2018). Telomere shortening also affects immune cells (Effros, 2011; Lin et al., 2015), and is a marker of aging hematopoietic stem cells (HSCs) (Morrison et al., 1996b). The telomerase complex is highly expressed and active in activated T cells and non-quiescent HSCs (Chiu et al., 1996; Morrison et al., 1996a), where it is required for HSC proliferation (Morrison et al., 1996a); however, telomere attrition is not fully prevented (Hsieh et al., 2017). Telomeres are protected by DNA–protein structures (the telomere complex) that counteract telomere shortening and maintain telomere length. Critical components of the telomere complex include telomerase reverse transcriptase (TERT) and telomerase RNA component (*TERC*) (Greider and Blackburn, 1985; Lingner et al., 1997), which cooperate to extend telomeres to maintain their repetitive TTAGGG nucleotide sequence (Blackburn, 2001; Townsley et al., 2014). The full control mechanisms that regulate telomere length comprise multiple layers of redundancy to ensure functionality in-case of system failure (Palm and de Lange, 2008). For example, TERT expression is up-regulated in response to *TERC* impairment (Vulliamy et al., 2001). Despite a comprehensive control network, genetic mutations in the telomere control machinery can occur and result in accelerated telomere shortening and severe disorders known as “telomeropathies.” Several telomeropathy models have been developed to better understand the disease mechanisms and discover new avenues for therapeutic intervention. For example, transgenic TERT-deficient mice exhibit accelerated telomere shortening associated with pathological abnormalities in the gut, extramedullar hematopoiesis in the spleen and liver and a skewed myeloid/erythroid ratio in the bone marrow (Strong et al., 2011).

Telomeropathies reported in human patients typically present with a wide range of clinical symptoms (Armanios and Blackburn, 2012; Holohan et al., 2014; Stanley and Armanios, 2015), the most severe being bone marrow failure (Ballew and Savage, 2013). Here, HSC transplantation is the main therapeutic option (Townsley et al., 2014), but long-term survival remains as low as 28% (Barbaro and Vedi, 2016). Telomeropathies associated with bone marrow failure syndromes, such as dyskeratosis congenita, aplastic anemia and myelodysplastic syndromes lack specific and effective therapies. In these cases, the most commonly used adjuvants are based on hormonal, immuno-suppressive, antioxidant or cytokine therapies (Fernandez Garcia and Teruya-Feldstein, 2014). The genetic mutations underlying autosomal dominant dyskeratosis congenita are well understood, as they typically affect the expression of the most integral components of the telomere complex — *TERC* (Mitchell et al., 1999; Vulliamy et al., 2001) or TERT. Here, *TERC* deficiency and deregulated telomere attrition results in loss of HSC renewal and potentially lethal bone marrow failure (Wong and Collins, 2006).

The impact of *TERC* impairment on hematopoiesis and the immune system has also been reported. Mice lacking *TERC* are more susceptible to the toxic effects of lipopolysaccharide than wild-type mice, due to increased chromosome instability in splenocytes and macrophages (Bhattacharjee et al., 2010). In corroboration with these findings, over-expression of TERT in embryonic stem cells provides a growth advantage and facilitates hematopoietic differentiation (Armstrong et al., 2005). A study using a reversible telomerase knockout mouse model found a direct link between TERT activity, telomere shortening and defective erythropoiesis (Raval et al., 2015). A normal phenotype could be re-established upon reactivation of telomerase. Finally, patients with dyskeratosis congenita exhibit immune impairments, including lymphopenia and elevated expression of senescence-associated (SA) markers, such as CD57, and a higher apoptosis rate compared to healthy subjects (Knudson et al., 2005). Surprisingly, non-telomeric roles for the telomerase complex have also been described in stem cells, especially the direct regulation of the Wnt differentiation-associated pathway mainly within the hematopoietic compartment (Park et al., 2009), but these findings are controversial (Strong et al., 2011). Furthermore, Yehuda et al. (2017) compared the expression and activity levels of DNA bound and cytoplasmic TERT in human fibroblasts showing that both fractions were dropping the expression and activity in senescent cells, although the diminishing was significantly more prominent in the cytoplasmic fraction of TERT. This leads to speculations that telomeric and non-telomeric functions of *TERC* during senescence are regulated independently (Yehuda et al., 2017).

Although bone marrow failure in telomeropathies is well described, we do not have a deep understanding of the underlying molecular mechanisms and the impact on specific immune-cell subsets. Here, we focused on the impact of dyskeratosis congenita on hematopoiesis and the immune functions of leukocytes. To detail the molecular processes underlying the loss of hematopoiesis, we generated genetically engineered human induced pluripotent stem cells (iPSCs) with shRNA-mediated *TERC* knock down. We then compared the telomerase activity, telomere length and other markers of cellular senescence with iPSCs expressing functional *TERC*, and assessed the differentiation capacity of HSCs and downstream myeloid cells. We anticipate that such iPSC-based disease models will further our understanding of the biology of dyskeratosis congenita and will be a valuable tool for investigating future therapies for patients with bone marrow failure due to defined telomerase deficiencies.

## Materials and Methods

### Human iPSC Culture

Undifferentiated human iPSCs (DF19-9-7T from WiCell) (Yu et al., 2009) were maintained in mTESR-1 (Stemcell Technologies) media on hES-qualified matrigel-coated tissue-culture plates (Thermo Fisher Scientific, Nunclon 140685), according to the manufacturer’s instructions. The cells were fed every day by replacing the whole media with fresh media, and were passaged using TrypLE Select (Invitrogen) when reaching ∼80% confluence (once a week) according to the single-cell iPSC culture method.

### Hematopoietic Differentiation

iPSCs were differentiated into CD34+ HSCs using protocols based on the use of embryonic bodies (EB) (Ng et al., 2008b; Jose et al., 2018). In brief, to generate spin EBs, TrypLE-adapted single cell iPSCs were cultured at ∼60–70% confluence, dissociated using TrypLE and then filtered through a 70-μm sterile filter to remove any clumps. Cells were seeded (3,000 cells per well in 100 μl) in a 96-well plate (Thermo Fisher Scientific Nunclon Sphera 96U-well ultra-low attachment plate 174929) or in AggreWell 800 plates (Stemcell Technologies 34815) with bovine serum albumin (BSA)—polyvinyl alcohol (PVA) essential lipids medium (BPEL) containing stem cell factor (SCF, 40 ng/ml), vascular endothelial growth factor (20 ng/ml), and bone morphogenic protein 4 (20 ng/ml). All growth factors were purchased from PeproTech. The media was prepared as previously described (Jose et al., 2018). In brief, 200 ml BPEL medium was prepared using 86 ml Iscove’s modified Dulbecco’s medium (Invitrogen), 86 ml F12 Nutrient Mixture with GlutaMAX I (Invitrogen), 5 ml 10% deionized BSA (Sigma-Aldrich), 10 ml 5% polyvinyl alcohol (Sigma-Aldrich), 20 μl 1 mg/ml linoleic acid (Sigma-Aldrich), 20 μl 1 mg/ml linolenic acid (Sigma-Aldrich), 0.4 ml Synthecol (Sigma-Aldrich), 1X α-monothioglyceral (Sigma-Aldrich), 5 ml protein-free hybridoma mix II (Invitrogen), 1 ml ascorbic acid (Sigma-Aldrich), 1% GlutaMAX I (Invitrogen), 1 ml insulin-transferrin-selenium solution (Invitrogen), and 1% penicillin/streptomycin (Invitrogen). Plates were then spin-aggregated at 280 × *g* for 5 min at room temperature and placed undisturbed in a 37°C incubator with 5% CO_2_. Cells were not removed for at least 3 days to ensure formation of spin EBs in the plates. Differentiation of CD34+ (CD34 APC eFluor 780, Invitrogen) Lin- (Human Hematopoietic Lineage Antibody Cocktail, eFluor 450, Invitrogen) HSCs was analyzed on day 13 of the culture.

### Myelopoietic Differentiation

At day 13 of spin EB differentiation, cells were transferred to a 24-well plate (six EBs per well) in BPEL without PVA and with the following growth factors: granulocyte-macrophage colony stimulation factor (GM-CSF, 40 ng/ml) interleukin (IL)-3 (20 ng/ml), IL-4 (40 ng/ml), SCF (40 ng/ml), and fms-like tyrosine kinase receptor-3 ligand (FLT3L, 20 ng/ml), all from Peprotech. After 12 days of culture, differentiation was confirmed by flow cytometry using CD11b+ CD13+ CD14+ antibodies (CD11b PE-Cyanine7, CD13 APC, CD14 PE, Invitrogen).

### THP-1 Cell Culture and Differentiation

The THP-1 pro-myelocytic leukemia cell line (Tsuchiya et al., 1980) was maintained in RPMI 1640 medium supplemented with 10% FBS, 1% glutamine and 1% Penicillin-Streptomycin. In some experiments, THP-1 cells were differentiated to a mature myeloid phenotype over 14 days in culture media containing the cytokines GM-CSF (40 ng/ml) and IL-4 (20 ng/ml) (both from Peprotech), changing half the media every 3 days.

### Plasmids and Lentiviral Constructs

The *TERC* knock down line in iPSCs (shTERC-iPSCs) and THP-1 cells (shTERC-THP-1) was generated by lentiviral transduction using lentiviral particles of *TERC* shRNA (sc-106994-V; Santa Cruz Biotechnology) or a scrambled shRNA control (sc-108080; Santa Cruz Biotechnology) used according to the manufacturer’s protocol. Stable cell lines were selected using puromycin (1 μg/ml).

### Colony Forming Units Assay

At day 13 of spin EB differentiation the differentiated EBs were dissociated using TrypLE, filtered through a 70-μm sterile filter and then the HSC population was enriched by magnetic sorting (MACS) using CD34 microbeads (Miltenyi Biotec). The CD34+ HSCs (50,000 cells/ml) were plated in methocult complete media (Stemcell technologies H4435 MethoCult Enriched medium). Colonies were counted and analyzed by FACS on day 14.

### Telomere Length T/S Analysis

A qPCR telomere length assay was performed as previously described (Cawthon, 2002) using telomere standard and a single copy gene (36B4). The average telomere length for each sample was calculated as a relative T/S ratio. PCR reactions were run on a Roche Lightcycler 480 and consisted of SYBR Green PCR Master Mix (Roche), forward and reverse primers, and 20 ng of DNA per reaction.

Primer sequences (5′→3′):

*Telomere standard:* 14x(TTAGGG)
*36B4 standard:* CAG CAA GTG GGA AGG TGT AAT CCG TCT CCA CAG ACA AGG CCA GGA CTC GTT TGT ACC CGT TGA TGA TAG AAT GGG
*TeloF:* CGG TTT GTT TGG GTT TGG GTT TGG GTT TGG GTT TGG GTT,
*TeloR:* GGC TTG CCT TAC CCT TAC CCT TAC CCT TAC CCT TAC CCT,
*36B4F:* CAG CAA GTG GGA AGG TGT AAT CC
*36B4R:* CCC ATT CTA TCA TCA ACG GGT ACAA

### TeloTAGGG Telomere Length Assay

A TeloTAGG telomere length assay (Roche #12209136001) was performed according to the manufacturer’s description, using 2 μg DNA starting material per sample. The DNA was restriction digested to retain only the telomere strand and then separated by agarose gel electrophoresis. The gel was analyzed by southern blotting on a positive charged nylon membrane and visualized by chemiluminescence (Anti-Dig).

### TeloTAGGG Telomerase Activity Assay

A TeloTAGGG telomerase assay (Roche #12013789001) was performed according to the manufacturer’s description, starting with 500,000 cells. In brief, telomerase protein was extracted and then added to a PCR reaction with the template provided and the activity of telomerase (TERT) was estimated after hybridization. The data represent the ability to elongate the template, which is detected by ELISA.

### Gene Expression Assay

Genomic DNA and RNA were isolated by column separation using a DNeasy Blood & Tissue Kit (Qiagen 69504) and RNeasy Plus Micro Kit with gDNA elimination (Qiagen 74034), respectively. All qPCR assays were run using TaqManprobes and TaqMan^TM^ Gene Expression Master Mix (Invitrogen 4369016) in a Roche Lightcycler 480. TaqMan^®^ Gene Expression Assay probes used were: GAPDH (Hs02758991_g1), OCT4 (Hs04260367_gH), SOX2 (Hs01053049_s1), NANOG (Hs02387400_g1), KLF4 (Hs00358836_m1), TERC (Hs03454202_s1), TERT (Hs00972650_m1), GATA1 (Hs01085823_m1), GATA2 (Hs00231119_m1), C/EBPα (Hs00269972_s1), IRF8 (Hs00175238_m1), RUNX1 (Hs01021970_m1), and SPI-B (Hs00162150_m1).

### Hematopoiesis Gene Array

Total RNA was treated with DNase I (Qiagen) and purified using an RNeasy Mini Kit (Qiagen). A total of 1 μg RNA was then reverse transcribed using a First Strand Synthesis Kit (Qiagen) and subsequently loaded on to an human hematopoiesis and hematopoietic stem cell RT2 profiler array (Qiagen 330231-PAHS-054Z), according to manufacturer’s instructions. Qiagen’s online web analysis tool was used to produce a comparative scatter plot and the fold change was calculated by determining the ratio of shTERC mRNA levels to control values using the Δ*C*t method (2^-ΔΔC_t_^). The *P*-values were calculated based on a Student’s *t*-test of the replicate 2^-ΔΔC_t_^ values for each gene in the control group and the shTERC group. All data were normalized to an average of five housekeeping genes Gusb, Hprt, Hsp90ab1, GAPDH, and ACTB. The PCR conditions were as follows: hold for 10 min at 95°C, followed by 45 cycles of 15 s at 95°C and 60 s at 60°C.

### Phagocytosis and ROS Assay

A phagocytosis assay was performed using pHrodo Green Zymosan Bioparticle Conjugate (Invitrogen P35365), according to the manufacturer’s instructions. In brief, the pHrodo particles (0.5 mg/ml) were added to the day 12 iPSC-derived myeloid culture or THP-1 cells and incubated at 37°C without CO_2_ for 1h. A ROS assay was performed on a BD FACS CANTO II cytometer (BD Biosciences) using a CellROX deep red flow cytometry assay kit (Invitrogen C10491), according to manufacturer’s instructions. In brief, 5 μM CellROX reagent was added to the cells in Iscove’s Modified Dulbecco’s Medium and incubated for 1 h.

After incubation, the cells were washed with PBS, acquired (BD FACS CANTO II) and analyzed using FlowJo (Tree Star). Dead cells were eliminated using live-dead staining with fixable viability dye eFlour 450 (Invitrogen 65-0863) or eFlour 780 (Invitrogen 65-0865).

### Flow Cytometric Detection of β-galactosidase

Senescence-associated (SA) β-galactosidase was analyzed using Detectagene Green CMFDG lacZ kit (Invitrogen D2920) (Merino et al., 2011; Buendia et al., 2015), according to the manufacturer’s instructions. In brief, 500,000 THP-1 cells were loaded using hypertonic influx reagent, and 1 mM substrate reagent was added to the cells and incubated at 37°C. The cells and substrate mixture were incubated for 10 min, followed by hypotonic lysis solution for 2 min. The cells were then re-suspended in recovery media with propidium iodide (PI) viability staining. After incubation at 37°C for 30 min, the cells were acquired (BD FACS CANTO II) and analyzed using FlowJo (Tree Star).

### Apoptosis Assay

An apoptosis assay was performed using an Annexin V Apoptosis Detection Kit eFluor 450 (eBioscience 88-8006-74), according to manufacturer’s instructions. In brief, 1 × 10^6^ THP-1 cells (two conditions: 24 h LPS stimulation and 7 days starvation media without FBS) were suspended in 100 μl 1X binding buffer and then incubated with 5 μl Annexin suspension for 20 min at room temperature. The cells were washed twice in binding buffer and re-suspended in 200 μl binding buffer. Finally, 5 μl PI solution (Invitrogen 00-6990) was added 5 min prior to acquisition (BD FACS CANTO II) and the data were analyzed using FlowJo (Tree Star).

### Primary HSCs Telomerase Inhibition

Primary CD34+HSCs were isolated from cytokine-mobilized peripheral blood cells or from bone marrow aspirate by MACS using CD34+ microbeads (Miltenyi Biotec). The CD34+ HSCs (5000 cells/ml) were added to methocult media (Stemcell technologies H4435 MethoCult Enriched medium) and then exposed to the following conditions: (1) untreated, (2) 2 μM BIBR1532 (TERT inhibitor from Merck Millipore 508839) or (3) 2 μM BRACO19 (telomere-telomerase binding inhibitor from Sigma-Aldrich SML0560). Colonies were counted and analyzed by FACS on day 14 for myeloid, granulocytic and erythroid-megakaryocyte lineages (CD15 eFlour 450, CD235a Biotin, Invitrogen).

### Statistical Analysis

All data are expressed as the means ± standard deviations (SD). Horizontal bars in figures indicate the means. Statistical significance was calculated using a two-tailed Student’s *t*-test for a single or multiparametric comparison for most experiments and a two-way ANOVA for some experiments as specified in figure legends. The data reported are pooled from three to five independent experiments. Statistical analysis was performed with Prism 6 software (GraphPad Software). Significance symbols (*ns* = *p* > 0.05; ^∗^*p* ≤ 0.05; ^∗∗^*p* ≤ 0.01; ^∗∗∗^*p* ≤ 0.001; ^∗∗∗∗^*p* ≤ 0.0001).

## Results

### *TERC* Depletion Reduces iPSC Hematopoietic Differentiative Capacity

The molecular mechanisms that underlie deficiencies in hematopoiesis, and ultimately bone-marrow failure in dyskeratosis congenita are unknown. Associations have been made between telomerase function, telomere length and clinical pathology, but a direct mechanistic link has not been shown. To address this issue, we established an *in vitro* cellular model of dyskeratosis congenita, in which we depleted a known causative gene, *TERC* in human iPSCs. We then used this cellular model to recapitulate the changes in hematopoietic differentiation that occur during disease progression.

**FIGURE 1:**
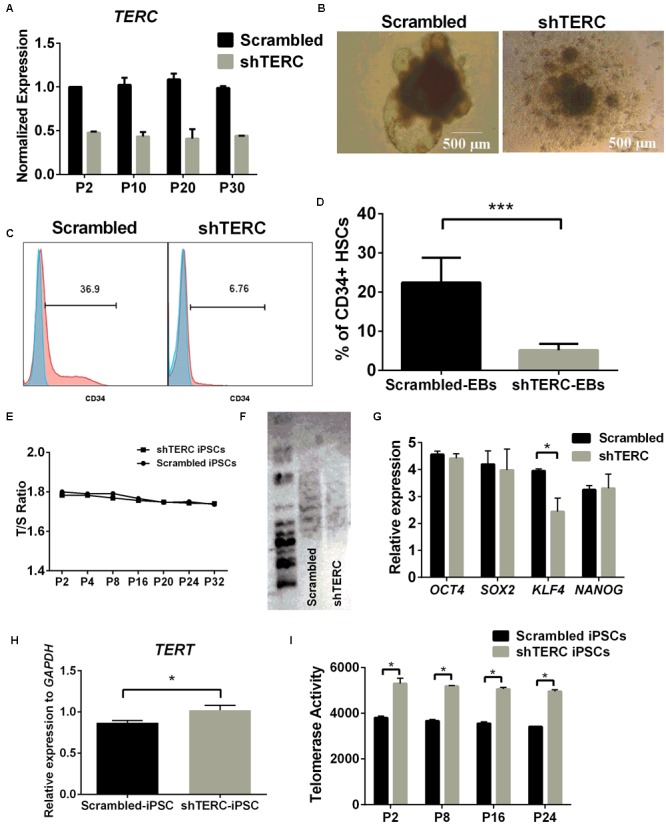
*In vitro* hematopoiesis of shTERC human induced pluripotent cells (iPSCs). **(A)** *TERC* mRNA expression (determined by qRT-PCR) was down-regulated by ∼50% in the knockdown (shTERC) compared to shRNA control (Scrambled) cells from passage (P) 2 – P30. **(B)** Bright field image of iPSC-derived day-7 hematopoietic embryonic bodies (EBs). shTERC shows disrupted architecture compared to the scrambled control. **(C)** FACS analysis showing day-13 shTERC EBs have reduced CD34+ hematopoietic stem cell (HSC) differentiation compared to scrambled control. **(D)** Proportion of CD34+ HSCs cells differentiated from shTERC-iPSCs compared to scrambled control EBs. Data are representative of seven experiments (*n* = 7). **(E)** Telomere length analysis by qPCR shows no significant decrease in the telomere length between shTERC and scrambled control. **(F)** TeloTAGGG telomere length assay showing no length variation between shTERC and scrambled control. **(G)** qPCR expression analysis of key pluripotency markers *OCT4, SOX2, KLF4, NANOG* in shTERC compared to scrambled control (*n* = 3, *p* < 0.05). Only KLF4 was significantly down regulated. **(H)** qPCR gene expression analysis showing significantly upregulated TERT expression in the shTERC iPSCs compared to control (*n* = 3, *p* < 0.05). **(I)** Roche TeloTAGGG Telomerase PCR elisa plus assay showing significantly higher telomerase enzyme (TERT) activity in the shTERC iPSCs compared to control (*n* = 3, *p* < 0.05).

Human iPSCs were transduced with *TERC* shRNA using a lentiviral delivery system to permanently deplete *TERC* expression. We achieved ∼50% down-regulation of *TERC* mRNA in shTERC-iPSCs compared to control iPSCs (scrambled shRNA) (**Figure 1A**). Assessment of the *TERC* mRNA expression levels between passages 2 and 30 showed that the effect of shRNA knockdown was stable (**Figure 1A**). An adopted protocol to derive HSCs from iPSCs was then used to analyze the hematopoietic differentiation capacity of shTERC-iPSCs (Ng et al., 2008a; Tian and Kaufman, 2008; Jose et al., 2018). We observed that shTERC-iPSCs lack the ability to form embryonic bodies (EBs) with a proper architecture at day 7 of differentiation compared to control iPSCs (**Figure 1B**). The capacity to differentiate into CD34+ (hematopoietic) cells, as measured by fluorescence-activated cell sorting (FACS), was also strongly affected in shTERC-iPSCs compared to controls (**Figures 1C,D**). Surprisingly, down-regulated TERC expression did not increase telomere attrition in shTERC-iPSCs, as shown by the equivalent T/S ratio of telomeres length between shTERC iPSCs and control iPSCs from passage 2–32 (**Figure 1E**). Telomere length analysis by TeloTAGGG assay also showed that TERC down-regulation did not lead to telomere shortening, as both control and shTERC showed the same telomere length (**Figure 1F**). To understand the pluripotent capacity of these cells, we analyzed the mRNA expression levels of the major pluripotency markers, *OCT4, SOX2, KLF4, NANOG*. Only *KLF4* mRNA was significantly down-regulated in shTERC iPSCs (**Figure 1G**). These data suggest a link between the telomerase and KLF4. Indeed, others have reported of crosstalk between KLF4 and telomerase; for example, the TERT promoter is activated by KLF4 and KLF4 has an important role in maintaining telomerase activity (Wong et al., 2010; Hoffmeyer et al., 2012). Interestingly, we observed increased *TERT* mRNA expression in shTERC cells and increased TERT activity (**Figures 1H,I**), despite reduced *KLF4* expression.

Taken together, these data show that *TERC* down-regulation results in imbalanced telomerase expression and activity in human iPSCs. While we did not observe increased telomere attrition in shTERC cells, there was a notable effect on the cellular capacity of iPSCs to differentiate into HSCs. This effect was observed despite normal expression of pluripotency markers (with the exception of KLF4) and a normal *T*/*S* ratio of telomere length. The lack of telomere attrition in shTERC cells could also be because of telomerase independent alternate lengthening of telomere (ALT) mechanisms that are shown to be exhibited by pluripotent stem cells (Huang et al., 2011). Furthermore down-regulation of *TERC* expression caused a significant increase in *TERT* mRNA expression and telomerase (TERT) activity. These data suggest that the iPSC differentiation impairment is not dependent on telomere length itself but could be an unrelated function of telomerase activity (i.e., non-telomeric activity of the telomerase complex).

### *TERC* Down-Regulation Impairs Myelopoiesis and Monocytic Cell Function

**FIGURE 2:**
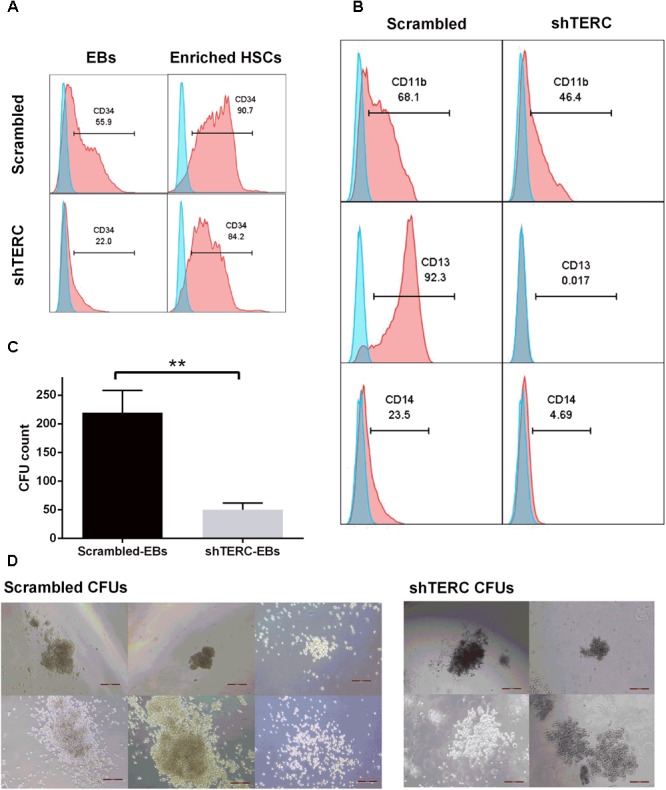
*In vitro* myelopoiesis of shTERC human induced pluripotent cells (iPSCs). **(A)** MACS enrichment of CD34+ HSCs from iPSC-embryonic bodies (EBs) in shTERC and shRNA control (Scrambled). **(B)** FACS analysis of shTERC-iPSC-derived hematopoietic stem cells (HSCs) showed reduced CD13+ CD11b+ myeloid differentiation potential compared to scrambled control, and subsequent development of CD14+ monocyte-like cells. **(C)** Colony formation unit (CFU) assay count. shTERC EBs formed a significantly lower number of blood colonies compared to scrambled control (*n* = 5, *p* < 0.01). **(D)** CFU assay bright-field images (Scale bar represents 100 μm; 10× magnification). Scrambled control EBs formed all six colony types while shTERC generated only four colony types.

Although the ability to differentiate into CD34+ HSCs was clearly affected in shTERC-iPSCs, we wanted to test whether differentiated HSCs could further progress into myelopoiesis. In order to achieve similar starting conditions in cellularity with control HSCs, we used magnetic separation to enrich the HSCs that developed into EBs (**Figure 2A**). Equal numbers of CD34+ HSCs derived from shTERC-iPSCs and shRNA scrambled controls were further differentiated in myelopoietic culture media for 7 days, and their differentiation was assessed by flow cytometry using major markers of myeloid differentiation (C11b, CD13, and CD14) (**Figure 2B**). While the majority of control progenitors expressed CD11b (68.1%), CD13 (92.3%), and CD14 (23.5%), the differentiation of shTERC-iPSC-derived HSCs was weak, as reflected by the markedly lower expression levels of CD11b (46.4%), CD13 (0.017%), and CD14 (4.69%). To further understand the hematopoietic capacity of CD34+ HSCs, the cells were seeded in semi-solid media for growth and enumeration of hematopoietic progenitor cells and the number of colony forming units was assessed after 14 days (**Figure 2C**). shTERC-iPSC-derived CD34+ HSCs gave rise to colonies of a similar size and shape to the scrambled control (**Figure 2D**), but the shTERC cells produced a significantly lower number of colonies (**Figure 2C**).

**FIGURE 3:**
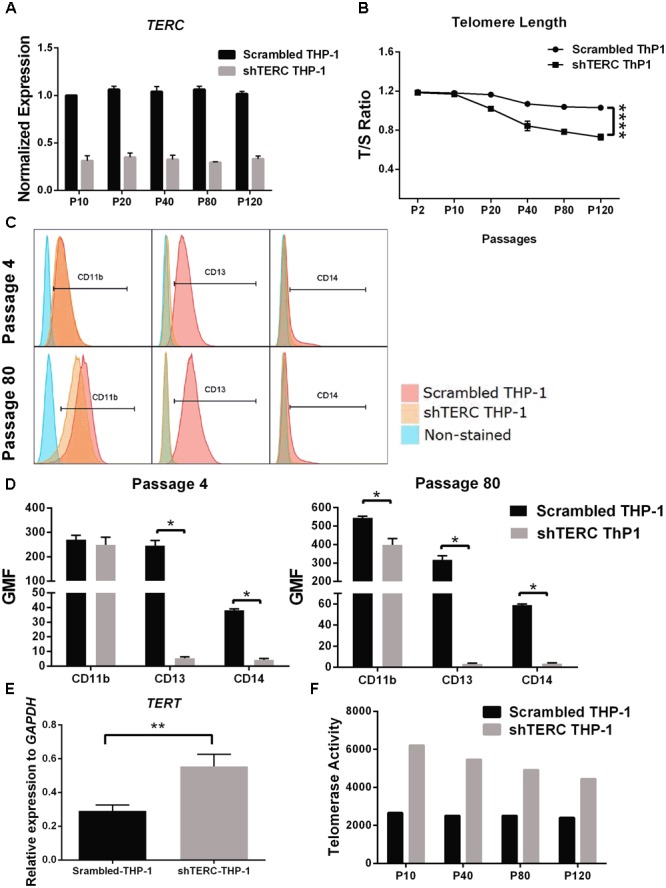
Monocyte-differentiation of shTERC THP-1 cells. **(A)** qPCR gene expression analysis of TERC in shTERC-THP-1 and scrambled control cells. *TERC* was robustly down-regulated in shTERC THP-1 cells from passage (P) 10 to P 120 (*n* = 3). **(B)** Telomere length assay showed reduced telomere length in shTERC THP-1 cells compared to scrambled control, and a tendency toward decreased telomere length with increasing passage number (*n* = 3, *p* < 0.0001, two-way ANOVA). **(C)** FACS analysis showed no CD13+ CD14+ monocyte differentiation by shTERC THP-1 cells compared to scrambled control cells, but no difference for CD11b myeloid marker expression over early and late passages. **(D)** shTERC-THP-1 at early passage 4 and late passage 80 both show significantly lower expression of CD13 and CD14 surface markers compared to control, while a significant reduction of CD11b was observed only at the latter passage (*n* = 3, *p* < 0.05). **(E)** Significantly up-regulated TERT gene expression was shown in shTERC THP-1 cells compared to control (*n* = 3, *p* < 0.01). **(F)** Roche TeloTAGGG telomerase activity assay showing increased telomerase enzyme (TERT) activity in the shTERC THP-1 line compared to control.

We next aimed to validate the iPSC data in a monocytic cell line (THP-1), to understand whether the telomerase imbalance caused by *TERC* down-regulation affects also later stages of differentiated myeloid cells. This validation in late passage numbers of THP-1 cells is required to compensate for the limitation of iPSCs to be passaged indefinitely as higher passages tend to reduce their pluripotency, reduce proliferation and undergo random spontaneous differentiation (Kim et al., 2010). We transduced THP-1 monocytic cells with the same *TERC* shRNA and tested myeloid differentiation. Here, ∼75% *TERC* down-regulation was achieved (shTERC-THP-1 cells) compared to control THP-1 cells (scrambled shRNA) (**Figure 3A**). In contrast to the iPSC model, down-regulation of *TERC* did lead to increased telomere attrition but only from passage 20, as measured by *T*/*S* ratio of telomeres length (**Figure 3B**). We measured the differentiative capacity of these shTERC-THP-1 cells and found that they failed to differentiate into a more mature myeloid phenotype that robustly expresses CD11b, CD13, and CD14 (**Figures 3C,D**). Importantly, the onset of impaired differentiation was observed as early as passage 4 after transduction, when the telomere length was not yet affected, but this phenotype was still evident at passage 80 (**Figures 3C,D**). Similarly to the iPSC, line, the down-regulation of *TERC* resulted in a significant increase of *TERT* mRNA expression and telomerase (TERT) activity (**Figures 3E,F**).

### *TERC* Deficiency in iPSC-Derived Myeloid Cells Affects the Expression of Transcription Factors Controlling Myelopoiesis

**FIGURE 4:**
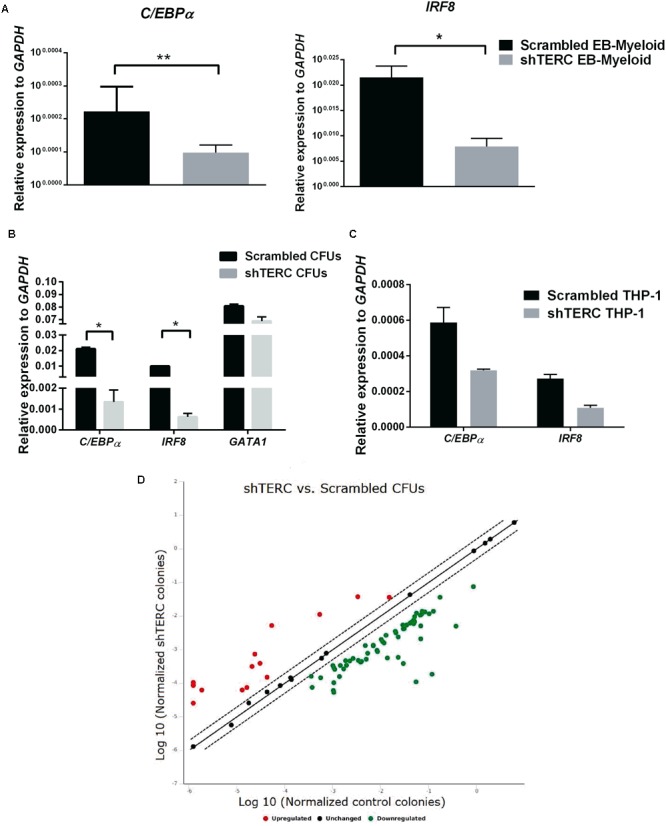
Expression of myeloid development factors (MDFs) by shTERC immune cells. **(A)** *C/EBPα* and *IRF8* mRNA expression in day 12 stage 2 embyronic bodies (EBs; myeloid state) showed significant down-regulation in shTERC cells compared to scrambled controls (*n* = 3, *p* < 0.01). **(B)** *C/EBPα* and *IRF8* mRNA was down-regulated in MACS-enriched EB-hematopoietic stem cell-derived CFUs (*n* = 3, *p* < 0.05). **(C)** *C/EBPα* and *IRF8* mRNA expression was also reduced in shTERC THP-1 cells compared to scrambled controls (*n* = 2). **(D)** RT-Profiler of hematopoiesis from induced pluripotent stem cell-EB-derived methocult colonies: shTERC vs. scrambled control showed a significant down-regulation of myeloid lineage associated genes and up-regulation of lymphoid-specific genes, including surface markers and cytokines (*n* = 3).

To understand which factors could change the potential of shTERC iPSCs to differentiate into myeloid cells, we analyzed the expression of a group of known transcription factors involved in myelopoiesis (Rosenbauer and Tenen, 2007). By quantitative real-time PCR, we found that both *C/EBPα* and *IRF8* were significantly down-regulated during shTERC-iPSC differentiation to myeloid cells compared to control iPSCs (**Figure 4A**). Similar results were obtained when the expression of these transcription factors was analyzed in the colony forming units (CFU) from methylcellulose cultures (**Figure 4B**) and in shTERC-THP-1 cells (**Figure 4C**). To understand the TERC-dependent changes in gene expression, we compared shTERC versus control CFUs isolated from methylcellulose cultures using a human hematopoiesis RT-profiler PCR array (**Figure 4D** and **Table 1**). Here, the RT-profiler analysis of various hematopoiesis related gene functional groups showed that the majority of myeloid differentiation and myeloid phenotype-related genes were significantly down-regulated in the shTERC-iPSC-derived colonies compared to control. Specifically, these genes included transcription factors (*GATA2, GATA1, C/EBPe, RUNX1*), myeloid regulators and activators (*MMP9, NOTCH* signaling, *DLL1, STAT1*), surface receptors (*TLR4, TLR3, CD14, CD86*), and growth factors/cytokines (*CSF1, CSF2, FLT3L, KITLG*).

**Table 1:** Human hematopoietic stem cells and hematopoiesis RT^2^ profiler^TM^ PCR array showing the significant variation in mRNA expression by shTERC iPSC-derived colony forming units compared to scrambled control.

Gene symbol	shTERC-CFUs	Scrambled-CFUs	Fold change	*p*-value
	2ˆ(-Delta *C*t)			
**Cell surface/cell lineage markers**			
CD14	4.94E-03	3.70E-01	–74.89	6.64E-09
CD86	2.36E-03	2.32E-02	–9.8	1.23E-06
PECAM1	4.41E-04	4.01E-03	–9.11	6.40E-06
CD164	1.16E-02	1.03E-01	–8.92	4.50E-09
SFXN1	4.12E-03	2.98E-02	–7.23	2.83E-06
CCR1	5.38E-03	3.50E-02	–6.5	2.82E-05
KIT	1.31E-02	8.47E-02	–6.47	3.66E-07
CD1D	2.63E-04	1.69E-03	–6.42	1.31E-05
CD44	3.59E-02	1.73E-01	–4.82	4.26E-09
CD34	1.44E-04	5.54E-04	–3.85	1.47E-05
CD80	3.73E-02	3.30E-03	11.29	0.0016
CD8A	3.87E-04	3.06E-05	12.64	0.0006
CD2	1.11E-02	5.36E-04	20.68	0.0045
CD3D	1.11E-02	5.36E-04	30.41	0.0032
CD4	5.17E-03	5.37E-05	96.34	2.80E-05
**Cytokines and growth factors**			
CSF2	6.01E-05	1.03E-03	–17.07	0.0015
FLT3LG	9.40E-04	8.38E-03	–8.92	0.0054
KITLG	4.72E-04	3.44E-03	–7.28	0.0002
CSF1	7.59E-03	4.92E-02	–6.48	0.0028
VEGFA	1.13E-02	7.11E-02	–6.29	1.42E-08
IL6ST	3.48E-03	2.04E-02	–5.87	2.18E-07
IL10	2.57E-04	1.10E-03	–4.26	0.0001
IL31RA	1.58E-04	3.59E-04	–2.27	0.0001
IL25	2.51E-05	1.24E-06	20.21	0.0142
IL11	6.21E-05	1.84E-06	33.75	0.0081
IL12B	8.48E-05	1.24E-06	68.28	0.0146
IL2	8.82E-05	1.24E-06	71.01	0.0172
IL20	1.04E-04	1.24E-06	83.86	0.0318
**Cell cycle regulators**			
NOTCH2	6.62E-03	4.44E-02	–6.7	4.37E-06
STAT1	9.50E-03	5.08E-02	–5.35	1.36E-06
JAG2	7.44E-05	3.70E-04	–4.97	0.0023
**Blood cell activation**			
TLR3	1.08E-04	5.41E-02	–499.15	0.0006
TLR4	5.85E-04	2.32E-02	–39.67	4.16E-05
HDAC7	6.72E-03	5.15E-02	–7.67	8.02E-07
HDAC5	1.71E-03	1.10E-02	–6.45	8.17E-06
HDAC4	2.65E-03	1.28E-02	–4.81	2.42E-05
HDAC9	4.57E-04	2.27E-03	–4.97	0.0002
**Cell differentiation**			
SPP1	7.46E-02	8.64E-01	–11.58	1.64E-08
PF4	4.19E-04	3.82E-03	–9.13	1.45E-08
BLNK	1.56E-04	1.38E-03	–8.84	1.26E-05
TAL1	5.83E-03	4.92E-02	–8.44	8.77E-06
NCOA6	1.57E-03	1.17E-02	–7.46	7.11E-09
MAP4K1	3.38E-04	1.93E-03	–5.72	9.38E-09
CHST15	1.28E-03	7.07E-03	–5.5	5.85E-06
SOCS5	1.99E-03	1.03E-02	–5.17	2.07E-06
**Hematopoiesis regulators**			
DLL1	5.27E-05	1.06E-03	–20.16	7.71E-08
MMP9	5.03E-03	6.90E-02	–13.71	1.12E-07
FUT10	1.02E-04	1.04E-03	–10.2	0.0004
TRIM10	5.13E-04	5.00E-03	–9.74	1.79E-05
LMO2	1.37E-02	1.26E-01	–9.19	1.20E-07
ASH2L	4.19E-03	3.10E-02	–7.4	5.65E-08
STIM2	1.31E-03	4.80E-03	–3.67	2.34E-06
**Signaling molecules**			
FZD1	7.71E-04	5.36E-03	–6.95	1.18E-06
STAT1	9.50E-03	5.08E-02	–5.35	1.36E-06
STAT3	1.37E-02	7.34E-02	–5.35	2.41E-08
JAG1	1.34E-03	6.95E-03	–5.19	4.68E-06
PTPRC	4.63E-04	1.87E-03	–4.04	2.17E-05
APC	2.89E-04	1.04E-03	–3.61	0.0002
NOTCH4	3.32E-04	9.98E-04	–3	0.012
**Transcription factors and regulators**			
GATA2	1.84E-04	1.17E-01	–639.15	1.07E-08
CEBPE	3.84E-04	2.95E-02	–76.82	0.0047
GATA1	2.07E-03	6.79E-02	–32.75	1.50E-08
CEBPG	5.58E-04	1.37E-02	–24.65	0.0011
ETS1	8.63E-04	8.56E-03	–9.92	5.45E-07
CBFB	6.11E-03	4.10E-02	–6.71	1.66E-06
VAV1	3.16E-03	2.05E-02	–6.5	7.04E-08
RBPJ	1.05E-02	6.68E-02	–6.36	1.77E-07
ETV6	5.36E-03	2.93E-02	–5.48	2.87E-08
NOTCH1	5.39E-04	2.68E-03	–4.98	3.31E-06
RUNX1	1.21E-02	5.58E-02	–4.59	8.47E-06
LEF1	3.34E-04	1.43E-03	–4.28	0.0002

*The genes are grouped according to their function*.

In summary, shTERC CD34+ precursors have a decreased capacity to progress further into myeloid differentiation. ShTERC-THP-1 cells also exhibit perturbed myelopoiesis, but in contrast to the shTERC iPSC-dervied CD34+ precursors, also underwent accelerated telomere attrition. Loss of *TERC* expression leads to impaired hematopoiesis at the molecular level, as shown by differential expression of hematopoietic regulatory transcription factors and numerous differentially expressed genes associated with hematopoiesis. The down-regulation of myeloid regulators (such as *GATA2, MMP9*), surface receptors (*CD14, TLRs*) and associated growth factors (*CSFs, FLT3L*) by shTERC knockdown colonies demonstrates the loss of functional myeloid differentiation ability in the telomerase-imbalanced cells. The elevated lymphoid surface markers and increased lymphoid associated cytokine gene expression in a pro-myelopoiesis condition could be an indication of HSCs being skewed toward a lymphoid lineage.

### *TERC* Deficiency Impairs Cell Function and Promotes Apoptosis in iPSC-Derived Myeloid Cells and THP-1 Monocytic Cells

**FIGURE 5:**
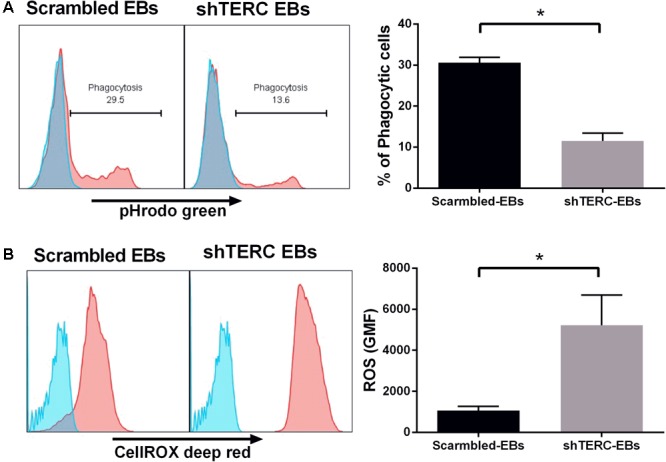
Functionality of shTERC human induced pluripotent cell (iPSC)-derived immune cells. **(A)** FACS-based phagocytosis assay (pHrodo Zymosan green particles) showed significantly lower phagocytic ability for shTERC-derived myeloid cells compared to scrambled control cells. The data are showing one representative experiment and mean of three independent experiments (*n* = 3, *p* < 0.05). **(B)** FACS–based cellular ROS assay (CellROX deep red) in the iPSC stage 1 day 13 embryonic bodies (EBs) showed significantly higher ROS production by shTERC-derived myeloid cells compared to scrambled controls. The data are showing one representative experiment and a mean of three independent experiments (*n* = 3, *p* < 0.05).

**FIGURE 6:**
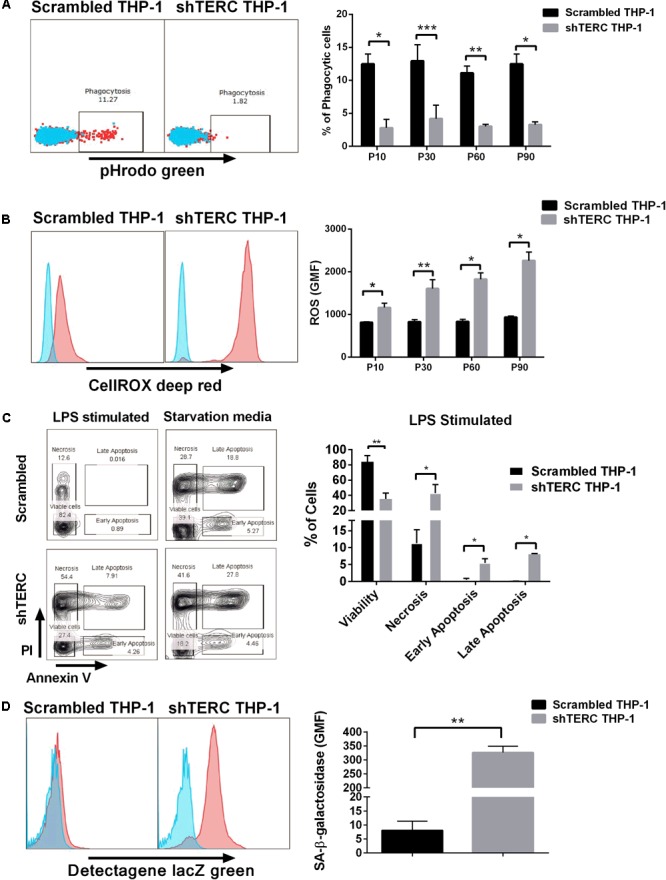
Characterization of shTERC THP-1 cells. **(A)** shTERC THP-1 cells from early and late passages showed reduced phagocytic ability compared to scrambled control cells (*n* = 3, *p* < 0.01, two-way ANOVA). **(B)** shTERC THP-1 cells from early and late passages were stimulated with lipopolysaccharide (LPS). shTERC THP-1 cells showed increased ROS production compared to scrambled controls (*n* = 3, *p* < 0.05, two-way ANOVA). **(C)** shTERC THP-1 cells showed a higher apoptotic rate compared to scrambled controls when LPS stimulated or starved for 7 days (*n* = 3, *p* < 0.05). **(D)** Senescence detection assay shows higher senescence-associated beta-galactosidase production by shTERC THP-1 cells compared to control (*n* = 3, *p* < 0.01).

Dyskeratosis congenita is mainly associated with loss of hematopoiesis, but data regarding functionality of myeloid cells and their progenitors are limited. Therefore, we addressed the phagocytic capacity and production of reactive oxygen species (ROS) in myeloid cells derived from shTERC-iPSCs (here called shTERC-EBs) and control cells (scrambled-EBs). The phagocytic capacity, as measured by amounts of cellular absorption of pHrodo zymosan particles, was clearly reduced in shTERC-EBs compared to scrambled-EBs (**Figure 5A**), indicating only a minor proportion of phagocytic cells in shTERC-EB sample. The levels of ROS after 24 h stimulation with LPS were also analyzed between shTERC-EBs and scrambled-EBs; here, shTERC-EBs had a significantly higher accumulation of ROS (**Figure 5B**). These findings were also reproduced in differentiated shTERC-THP-1 cells. Down-regulation of *TERC* expression resulted in a significant decrease in the proportion of phagocytic cells (**Figure 6A**) and increased ROS production in response to LPS in shTERC-THP-1 cells compared to control cells (**Figure 6B**). In addition, the ROS levels gradually increased with passage number (**Figure 6B**).

To understand how *TERC* depletion can influence phagocytic cell numbers, we analyzed the intrinsic cellular stress of shTERC-THP-1-derived myeloid cells by SA-β-galactosidase and apoptosis assay. Interestingly, lack of *TERC* lead to higher amounts of apoptotic cells (12%) 24 h after LPS stimulation (**Figure 6C**), while the shTERC-THP-1 showed higher SA-β-galactosidase production compared to control cells (**Figure 6D**).

These data show that *TERC* deficiency in myeloid-derived iPSCs and THP-1 cells leads to changes in several processes linked to cellular phagocytic function, proliferation and differentiation, such as apoptosis. Most importantly, higher production of ROS and SA-β-galactosidase clearly highlight a senescent cellular phenotype as a result of down-regulated *TERC* expression. These *in vitro* observed changes caused by telomerase imbalance may explain some of the processes associated with bone marrow failure in patients with dyskeratosis congenita.

### Diminishing Telomerase Activity With Small Molecule Inhibitors Impairs Myelopoiesis

**FIGURE 7:**
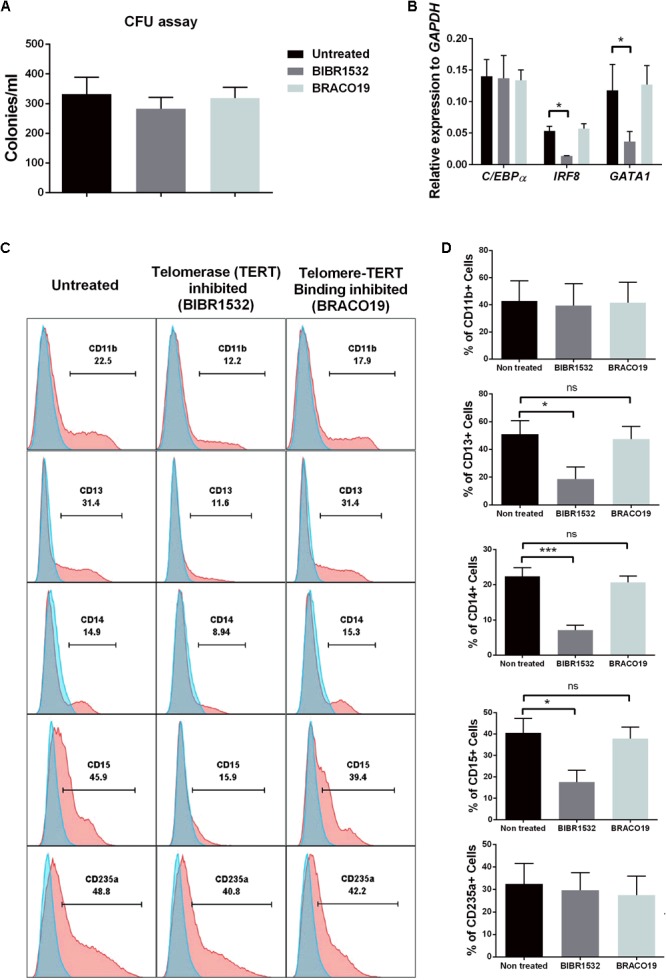
Influence of Telomerase (TERT) directly in differentiation of hematopoietic stem cells (HSCs). **(A)** Day 14 colony forming unit (CFU) colony count of primary CD34+ HSCs exposed to the following conditions: untreated, TERT inhibitor (BIBR1532) or Telomere-telomerase binding inhibitor (BRACO19) (*n* = 5). **(B)** *IRF8* and *GATA1* myeloid transcription factors were significantly down-regulated at the mRNA level in TERT-inhibited cells compared to non-treated and telomere-telomerase binding inhibited (*n* = 3, *p* < 0.05). No change in *C/EBPα* expression was detected between all three conditions. **(C)** FACS analysis of primary HSC-derived colonies showing reduced myeloid-granulocyte differentiation potential following TERT inhibitor treatment compared to non-treated and telomere-telomerase binding inhibitor treatment. No effect of TERT inhibition was found on the erythroid-megakaryocyte lineage (CD235a+). **(D)** TERT inhibition leads to significant reduction in expression of CD13, CD14, and CD15 surface markers compared to non-treated and telomere-telomerase inhibited cells (*n* = 5, *p* < 0.05). No effect was detected on CD11b and CD235a expression between all three groups.

We finally aimed to corroborate our findings based on shRNA silencing of *TERC* expression using two different telomerase inhibitors. To understand the influence of telomerase activity in hematopoiesis, we exposed cells to BIBR1532 [a specific inhibitor of TERT activity (Damm et al., 2001)] and BRACO19 [that blocks TERT binding to telomeres, while preserving the activity (Riou et al., 2002)]. Here, primary CD34+ hematopoietic precursors were isolated from bone marrow aspirates and cells were seeded in complete methylcellulose for differentiation in the presence or absence of the two inhibitors. No effect on the numbers of CFUs from CD34^+^-enriched cells in presence of inhibitors was found compared to untreated cells (**Figure 7A**). However, cells exposed to BIBR1532 expressed significantly lower amounts of *IRF8* and *GATA1*, but in contrast to the effects of shRNA knockdown of *TERC, C/EBPα* was unaffected (**Figure 7B**). The BRACO19 inhibitor had no effect on these transcription factors.

This direct action of inhibited telomerase activity (via BIBR1532 exposure) on the hematopoietic potential of progenitors was further confirmed by analyzing the phenotype of cells in colonies harvested from methylcellulose cultures. By flow cytometry, we found a significant impairment of BIBR1532-inhibited cultures to differentiate into CD13+, CD14+ (myeloid), and CD15+ (granulocyte) lineages (**Figures 7C,D**). Importantly we did not observe any significant changes in differentiation to CD235a^+^ cells, a megakaryocyte-erythroid lineage marker, suggesting the specific influence of telomerase activity on myeloid differentiation. Furthermore, none of the changes induced by the BIBR1532 telomerase inhibitor were observed using BRACO19, which prevents telomerase from binding to telomere sequences. These data clearly indicate specific, non-telomeric activity of telomerase.

These data are an important validation of our previous results based on shRNA-mediated *TERC* silencing during long-term culture. The ability to reproduce the phenotype using a telomerase inhibitor highlights the direct role of telomerase in regulating myeloid cell differentiation. These data suggest that it is not telomere attrition but telomerase function that is responsible, in part, for failed hematopoiesis and ultimately bone-marrow failure in telomeropathies.

## Discussion

Human telomeropathies comprise a wide spectrum of disorders, most of them associated with accelerated senescence and bone marrow failure. Augmented telomere attrition is a major molecular indicator of disease progression, but not all pathological symptoms — in particular bone marrow failure — can be explained by shortened telomeres. Here, we recapitulated hematopoietic differentiation *in vitro* using an iPSC model and shRNA-mediated *TERC* down-regulation to mimic the *TERC* impairment found in some human telomeropathies (Mitchell et al., 1999; Vulliamy et al., 2001). Partial *TERC* down-regulation limited the capacity of CD34+ HSCs to differentiate from iPSCs, but the expression of most pluripotent markers remained almost unchanged. To date, the role of TERC in controlling hematopoiesis has been controversial. A *TERC*^-/-^ mouse model showed that HSCs were able to prevent the DNA damage associated with shortened telomeres but remained quiescent due to cell-cycle arrest, which was associated with cellular senescence (Wang J. et al., 2014). Furthermore, a reduced number of bone marrow progenitors and CFUs in patients with dyskeratosis congenita compared to unaffected siblings has been reported (Marsh et al., 1992). Conversely, others have observed a strong skewing of HSCs isolated from older donors to the myelopoietic lineage due to accumulated DNA damage (Liang et al., 2005) or cytokine control (Ergen et al., 2012). The data from our iPSC model support the latter, that either *TERC* directly or a telomerase complex imbalance caused by *TERC* down-regulation interferes with HSC differentiation, but also imply that *TERC* mediates HSC differentiation independently of telomere shortening.

Goldman et al. (2008) showed a low frequency of CD34+ HSCs and reduced telomere length in patients with *TERC*-mutated dyskeratosis congenita compared to age-matched donors. Here, bone marrow failure in dyskeratosis congenita was considered to be caused by reduced HSC numbers due to their non-proliferating nature rather than a qualitative defect in their commitment to specific lineages (Goldman et al., 2008). However, only the variation in number for each blood lineage compartment was screened, whereas the functionality of telomerase-impaired immune cells was not assessed. Clonal hematopoiesis of patients with dyskeratosis congenital could be a major reason for the reduced functionality exhibited by telomerase-impaired immune cells. For example, Perdigones et al. (2016) demonstrated that clonal hematopoiesis is a common phenomenon in affected patients, and these somatic changes could lead to alterations in cellular characteristics including induction of malignancy.

Telomere regulatory mechanisms are robust, as illustrated by experiments showing that leukemic cells can recover from inhibitor-mediated telomere shortening (Delhommeau et al., 2002). Thus far, changes in telomere length and aging-related pathologies have been only correlative; indeed, also in dyskeratosis congenita the telomere length has not been proven to be the direct molecular mechanism underlying the clinical pathology (Nelson and Bertuch, 2012). Telomere complex impairment is one of the first explanations for bone-marrow failure in dyskeratosis congenita, but several groups have highlighted a possible role for bone marrow mesenchymal stem cells in disease pathology. Specifically, mesenchymal stem cells lacking *TERC* are unable to sufficiently support healthy hematopoiesis (Balakumaran et al., 2015; Chen et al., 2015). Nevertheless the role of specific members of the telomerase complex in telomeropathy progression remains inconclusive. One study reported a significantly shorter telomere length and impaired hematopoiesis in iPSCs derived from patients with severe aplastic anemia, compared to healthy controls (Melguizo-Sanchis et al., 2018). Loss of self-renewal has also been identified in iPSCs derived from patients with dyskeratosis congenita in which dyskerin is mutated (Batista et al., 2011). Despite these advances, none of the reports addressed the role of the telomerase complex in hematopoietic differentiation.

The emerging tools allowing reprogramming of somatic cells from patients with dyskeratosis congenita into *TERC*^-/-^ patient-derived iPSCs have provided controversial results. However, studies have shown that reprogrammed iPSCs isolated from those with *TERC*-mutated dyskeratosis congenita can restore telomere maintenance due to up-regulated levels of telomerase found in the pluripotent state (Agarwal et al., 2010; Winkler et al., 2013). Others have reprogrammed fibroblasts isolated from patients with dyskeratosis congenita into patient-derived iPSCs and then performed mutation correction by CRISPR/Cas9 technique (Fok et al., 2017). Of note, such iPSC reprogramming frequently allows cells to extend the telomeres length (Huang et al., 2011; Winkler et al., 2013), therefore it is challenging to dissect the level of molecular control of telomeres length versus the activity of telomerase. Another caveat is that pluripotent stem cells undergo changes in telomerase activity during lineage differentiation process (Huang et al., 2014). While these findings have a great therapeutic potential, it complicates the use of patient-derived iPSCs as a model helping to understand the mechanism of the disease.

Telomere complex compensates for the lower expression of its components, in particular TERT was shown to be upregulated in *TERC*^-/-^ mice or in *TERC* mutated patients (Vulliamy et al., 2001). This phenomenon recapitulates in shTERC model showing *TERT* mRNA expression elevated upon down-regulation of *TERC* and incidentally shows higher telomerase activity. However, tagging the C-terminus of TERT by Human influenza hemagglutinin showed that telomerase activity does not necessarily results in telomere maintenance, but could link extra-telomeric activities to increasing cellular life span (Ouellette et al., 1999).

The contribution of extra-telomeric activity of telomerase is now being intensively studied. Novel roles for the telomerase complex unrelated to telomere maintenance, but rather the direct crosstalk between telomerase and various inflammatory signals, have been suggested (Jose et al., 2017). *TERC* knockdown induces the intrinsic apoptotic pathway in primary T cells via a molecular mechanism independent of telomere length or damage (Gazzaniga and Blackburn, 2014). Here, over-expression of active TERT results in apoptosis only in the absence of *TERC*, thus illustrating the important crosstalk between *TERC* and TERT as well as the extra-telomeric functions of *TERC* (Gazzaniga and Blackburn, 2014). Another study found that retroviral expression of human TERT in a cytokine-dependent, human hematopoietic progenitor cell line (TF-1) permits cell survival after cytokine withdrawal, but not unlimited replication (Li et al., 2006). This TERT-mediated effect on cell survival resulted in an accumulation of cells in G1 phase that operated via autocrine expression of IL-3 and activation of the p53/p21 pathway, indicating a non-telomere dependent role of TERT in autocrine cytokine secretion. These reports of extra-telomeric functions of *TERC* (and TERT) corroborate our finding of impaired haematopoiesis and increased apoptosis in our shTERC iPSC model, in which telomere shortening was absent.

The extra-telomeric roles of both *TERC* and TERT in hematopoiesis have been demonstrated in zebrafish models (Alcaraz-Perez et al., 2014), but data obtained from human or animal models remains limited. Genetic depletion of *TERC* in the zebrafish results in impaired myelopoiesis, despite normal HSC development (Alcaraz-Perez et al., 2014). Genetic analysis showed that *TERC* modulates the myeloid-erythroid fate decision by controlling the levels of the master myeloid and erythroid transcription factors SPI1 and GATA1. The alteration in SPI1 and GATA1 levels occurs through stimulation of GCSF and MCSF promoters by *TERC*, illuminating the non-canonical roles of *TERC* in these pathways (Alcaraz-Perez et al., 2014). The hematopoietic transcription factors and their influence by *TERC* are key findings to support our results demonstrating the down-regulation of certain transcriptions factors in shTERC cells and their subsequent reduction in hematopoiesis. Similarly, knockdown of TERT in zebrafish leads to depletion in blood cell count, as hematopoietic cell differentiation is impaired, whereas other somatic lineages remain morphologically unaffected. However, only the reverse transcriptase motifs of TERT are crucial for rescuing complete hematopoiesis, as the telomerase RNA-binding domain of TERT needed for telomere elongation is not required. This effect demonstrates the non-canonical pathway of TERT to modulate HSC differentiation and proliferation (Imamura et al., 2008). The mechanism underlying this effect may be similar to that in humans, as our data using a TERT inhibitor on BM-HSCs produced similar results.

Our analysis of pluripotent marker expression detected a decrease in *KLF4* mRNA in shTERC iPSCs. Although this finding only shows a correlation with changes in differentiation capacity, others have shown a direct crosstalk between the telomerase complex and KLF4 expression (Wong et al., 2010; Hoffmeyer et al., 2012). We additionally found that the reduced capacity of shTERC-iPSCs to differentiate into HSCs also impaired myelopoiesis. The onset of myelopoiesis can be recognized by changes in expression of key transcription factors (Rosenbauer and Tenen, 2007), namely *IRF-8, IRF-4, CEBP/a*, or *GATA-1* that drive differentiation toward myeloid, lymphoid or erythroid lineages. Consistently, we found that our shTERC-iPSC-derived myeloid cells have impaired *IRF-8, GATA-1*, and *CEBP/a* RNA expression. Interestingly, some of these transcription factors up-regulate telomerase activity through TERT expression (Hrdlickova et al., 2009), but CEBP/a regulates myeloid differentiation through *TERC* activation (Kumar et al., 2013). As such, we propose that differential expression of the key transcription factors that drive myeloid cell differentiation may contribute, in part, to the observed poor myeloid differentiation in our experimental setting. We also assessed the consequences of lack of *TERC* expression in THP-1 monocytes *in vitro*, to determine whether there is a direct effect of *TERC* expression on myelopoiesis. Again, *TERC* knockdown impaired differentiation of myeloid cells, and the differentiated myeloid cells exhibited decreased phagocytic capacity, increased ROS production and SA-β-galactosidase production. These functions have been frequently associated with cellular senescence (Taniguchi Ishikawa et al., 2012; Pereboeva et al., 2013; Shao et al., 2013). However, here we observed telomere shortening in the differentiating THP-1 cells only at late passages, while the impaired myeloid differentiation and senescence associated functions are seen even at early passages, which implies a direct influence of telomerase activity on the expression of key senescence markers and myeloid cell function in THP-1 cells.

*TERC^-/-^* mouse models exhibit an increased proportion of myeloid cells and impaired red blood cell and B-cell development in older animals (Ju et al., 2007; Chen et al., 2015). A link between intestinal infection and inflammation with impaired telomerase activity has also been shown in these animals. In contrast to our findings *in vitro*, normalization of myeloid cell numbers in *TERC^-/-^* mice following antibiotic treatment had no effect on the HSC compartment, suggesting that the effect of loss of *TERC* on myelopoietic changes is indirect (Chen et al., 2015).

Direct roles of telomerase activity on the immune system have been shown; for example, inflammatory stimuli can induce telomerase activity in macrophages (Gizard et al., 2011). Short telomeres in human leukocytes are a marker of immunosenescence (Akbar and Vukmanovic-Stejic, 2007; Plunkett et al., 2007; Deelen et al., 2014). When peripheral blood mononuclear cells were isolated from patients after autologous and allogeneic HSC transplantation, the recipient HSC telomere length was found to be shorter than the donor HSC telomere length (Wynn et al., 1998, 1999; Lee et al., 1999; Akiyama et al., 2000). Telomere loss has also been confirmed in individual blood cell types following allogeneic bone marrow transplantation, being the most conclusive in chronic graft-versus-host disease (Baerlocher et al., 2009). Some have suggested that such major telomere loss within particular HSC compartments may be responsible for the high rate of telomere shortening in peripheral blood lineages where committed progenitors readily replenish the recipient blood cell count (Wynn et al., 1999). Even transplants using peripheral blood progenitor cells rather than bone marrow are able to re-establish the blood cell count and abrogate replicative stress to some degree. But despite this effect, telomere shortening reaches equivalent levels in neutrophils and T cells when comparing recipients and donors in mobilized blood versus bone marrow transplantation.

Many of the experimental models used to address the role of telomere shortening in human aging and disease have been very limited in terms of providing mechanistic data. This is due, in part, to a lack of suitable models and the correlative nature of most clinical observations. *In vitro* cellular models can provide a mechanistic understanding as to how the telomerase complex functions, but the conclusions toward aging-related pathologies are also limited due to lack of model complexity and different time perspectives. Data generated in genetically engineered murine models are often not sufficient to explain the aging mechanism in humans, given that mouse telomeres can be up to 10 times longer than their equivalent human sequences despite a much shorter animal lifespan (Calado and Dumitriu, 2013). Nevertheless, using a *TERC*^-/-^ murine model importantly showed that lack of *TERC* results in telomere shortening, a higher incidence of tumor formation (Blasco et al., 1997) and accelerated aging (Samper et al., 2001).

## Conclusion

Telomere attrition is one of the hallmarks of aging and cellular senescence (Lopez-Otin et al., 2013). The detrimental effects of accelerated telomere attrition, leading to apoptosis, DNA damage or even organ failure, have been demonstrated (Armanios et al., 2009). On the other hand, some of the newly shown non-telomeric activities of telomerase complex, which are also affected by mutations in some of telomerase complex members, are also able to explain some of the aspects of dyskeratosis congenita pathology. Different methodological approaches, such as murine models or iPSCs have been recently used to dissect the exact mechanism underlying telomeropaties pathology. Our study used innovative cellular models to conclude that telomerase activity has a direct role in hematopoiesis. Using inhibitors of the telomerase complex and telomerase-telomere binding, we could show that a non-telomeric function of telomerase has a major role in activating expression of senescence markers. In addition, we provide evidence as to how telomerase activity directly impacts myeloid cell development and function. Finally, we found that myeloid cells with impaired telomerase express elevated senescence markers. Future studies are now needed to further dissect the exact mechanisms of hematopoietic impairments and bone-marrow failure associated with telomeropathies and identify new therapeutic targets. Until new therapeutic strategies can be designed, the major curative option for patients with telomeropathies will remain bone marrow transplantation. Although bone marrow transplantation is initially effective in eliciting extensive HSC expansion upon engraftment, telomere attrition also occurs in donor cells (Lee et al., 1999; Akiyama et al., 2000; Baerlocher et al., 2009; Wang L. et al., 2014b). The need for new therapeutic interventions that support telomere maintenance in the context of bone marrow transplantation is alarming.

## Ethics Statement

This study was carried out in accordance with the recommendations of The Institutional Review Board of the University Hospital Brno with written informed consent from all subjects. All subjects gave written informed consent in accordance with the Declaration of Helsinki. The protocol was approved by The Institutional Review Board of the University Hospital Brno.

## Author Contributions

SSJ designed and performed the experiments, analyzed the primary and aggregated data, and wrote the manuscript. FT and PB performed some experiments. TK provided bone marrow aspirates and supported with clinical expertise. KB supervised some experiments. JF conceived and supervised the project, designed the experiments, secured funds, and wrote the manuscript.

## Conflict of Interest Statement

The authors declare that the research was conducted in the absence of any commercial or financial relationships that could be construed as a potential conflict of interest.
